# Can a standard dose of eicosapentaenoic acid (EPA) supplementation reduce the symptoms of delayed onset of muscle soreness?

**DOI:** 10.1186/1550-2783-9-2

**Published:** 2012-01-31

**Authors:** David Houghton, Gladys L Onambele

**Affiliations:** 1 Department of Exercise and Sports Science, Manchester Metropolitan University. Crewe Green Road, Crewe CW1 5DU, UK; 2 Institute for Cell and Molecular Bioscience, Framlington Place, Newcastle Upon Tyne, NE2 4HH, UK

**Keywords:** EPA, IL-6, resistance exercise and Delayed Onset Muscle Soreness

## Abstract

**Background:**

Unaccustomed exercise can result in delayed onset of muscle soreness (DOMS) which can affect athletic performance. Although DOMS is a useful tool to identify muscle damage and remodelling, prolonged symptoms of DOMS may be associated with the over-training syndrome. In order to reduce the symptoms of DOMS numerous management strategies have been attempted with no significant effect on DOMS-associated cytokines surge. The present study aimed to investigate the acute and chronic effects of a 2 × 180 mg per day dose of eicosapentaenoic acid (EPA) on interleukin-6 (IL-6) mediated inflammatory response and symptoms associated with DOMS.

**Methods:**

Seventeen healthy non-smoking females (age 20.4 ± 2.1 years, height 161.2 ± 8.3 cm and mass 61.48 ± 7.4 kg) were randomly assigned to either placebo (N = 10) or EPA (N = 7). Serum IL-6, isometric and isokinetic (concentric and eccentric) strength, and rating of perceived exertion (RPE) were recorded on four occasions: i-prior to supplementation, ii-immediately after three weeks of supplementation (basal effects), iii-48 hours following a single bout of resistance exercise (acute training response effects), and iv-48 hours following the last of a series of three bouts of resistance exercise (chronic training response effects).

**Results:**

There was only a group difference in the degree of change in circulating IL-6 levels. In fact, relative to the first baseline, by the third bout of eccentric workout, the EPA group had 103 ± 60% increment in IL-6 levels whereas the placebo group only had 80 ± 26% incremented IL-6 levels (P = 0.020). We also describe a stable multiple linear regression model which included measures of strength and not IL-6 as predictors of RPE scale.

**Conclusion:**

The present study suggests that in doubling the standard recommended dose of EPA, whilst this may still not be beneficial at ameliorating the symptoms of DOMS, it counter intuitively appears to enhance the cytokine response to exercise. In a context where previous in vitro work has shown EPA to decrease the effects of inflammatory cytokines, it may in fact be that the doses required in vivo is much larger than current recommended amounts. An attempt to dampen the exercise-induced cytokine flux in fact results in an over-compensatory response of this system.

## Introduction

Although exercise is generally shown to be beneficial, a bout of resistance exercise that an individual is unaccustomed to can result in a reduction in force generating capacity (RFGC) and post-exercise muscle soreness, commonly known as Delayed Onset Muscle Soreness or DOMS [1,2]. There is no known definitive cause of DOMS, although Lenn et al. [3] suggested that there are two concurrent mechanisms responsible. The initial mechanism for muscle damage occurs following unaccustomed exercise (predominantly eccentric contractions). The damage to muscle fibres ranges from alterations to a small number of macromolecules to large tears in the sarcolemma, basal lamina and in the surrounding connective tissue [4,5]. Following damage to skeletal muscle the secondary mechanism is a loss of intramuscular protein and the release of growth factors that modulate satellite cells activity, which begin the repair and regenerative process [4,5], as well as involving the production of biochemical end products including cytokines. Asmussen [6] indicated that these biochemical end products may affect nerve endings and activate nociceptors creating the sensation of muscle soreness. The functional impact of this muscle soreness was addressed by Graven-Nielsen et al. [7], who demonstrated that those experiencing muscle pain were unable to achieve maximum voluntary contractions (MVCs).

The structural damage to the contractile proteins and membranes within skeletal muscle signals the hypothalamic pituitary adrenal axis (HPA) to produce acute phase proteins in, and around, the damaged site. The production of acute phase proteins includes the production of cytokines, specifically those that initiate the incursion of lymphocytes, neutrophils and monocytes, which instigates the healing phase, thereby emphasising the importance of the cytokines produced [8,9]. Some of the cytokines produced include tumour necrosis factor alpha (TNF-α), interleukin-1 (IL-1), interleukin-6 (IL-6) and interleukin-10 (IL-10) [9]. These cytokines have been identified as pro-inflammatory cytokines due to the similarities with responses to trauma and infection when injected into humans [10]. IL-6 in particular, has been suggested to possess both pro - and anti-inflammatory properties and is therefore generally referred to as an inflammation responsive cytokine [11,12].

Northoff et al. [13] suggested that increases in IL-6 may be involved in the generation of acute phase inflammation post exercise. To date, research indicates that the substantially increased IL-6 both during and post resistance exercise, may be dependent on the intensity and nature of muscular contraction [2,14]. Similarly, Pedersen et al. [14] suggested that the level of DOMS experienced is linked to the quantity of IL-6 produced. Interestingly, the work of Pedersen et al. [14], and further research by Richards et al. [11] suggest that the IL-6 response experienced post exercise, may not be entirely beneficial nor necessary for muscle development. This has led to research on the effects of excessive levels of IL-6 both *in vivo *and *in vitro*. Bauman et al. [15] and Febbraio et al. [16] linked excessive levels of IL-6 to cancer and chronic inflammation in elderly individuals. Possible underlying mechanisms include a deleterious positive feedback loop of the hypothalamic-pituitary adrenal (HPA) axis and an increase in C-reactive protein (CRP) production. Yet, in contrast to aforementioned studies, Al-Shanti et al. [17] demonstrated *in vitro *that IL-6 in combination with TNF-α, promoted myoblast cell proliferation. Therefore, IL-6 appears to have both positive and negative effects associated with muscle repair and regeneration. It is unclear, however, at what point IL-6 levels may become detrimental. If an elevated IL-6 response in muscle damage is not essential for muscle development, then a reduction in IL-6 may positively impact recovery time from exercise, whilst simultaneously optimising performance. There is sufficient evidence to suggest that the cytokines produced post muscle damage are linked to DOMS [2,11,13,14]. Consequently in an attempt to reduce the cytokine response and DOMS following muscle damage, numerous management modalities such as massage, cryotherapy, stretching and ibuprofen have been tried though the evidence regarding either their benefit or otherwise is still not conclusive [3].

There is evidence to suggest that dietary supplements such as omega-3 containing fish-oil, specifically the polyunsaturated fatty acid 20:5n3 component (also commonly known as eicosapentaenoic acid or EPA), may be efficient at reducing the pro-inflammatory cytokines associated with inflammation [18,19]. Magee et al. [18] demonstrated *in vitro *that EPA inhibited the effects of TNF-α by reducing its apoptotic effects and enabling myogenesis, thus allowing optimal skeletal muscle cell differentiation from myoblasts into myotubes, a process which is key in the regeneration of muscle following damage. Complimentary evidence was provided *in vivo *by Matsuyama et al. [19] who worked with patients suffering from chronic obstructive pulmonary disease (COPD). COPD is characterised by chronic inflammation and pain in the throat and chest when breathing. Matsuyama et al. [19] treated patients for 24 months with EPA supplementation. With treatment, participants exhibited lower TNF-α levels and reported a reduction pain in comparison with baseline values. The findings from these two studies suggest a link between elevated levels of pro-inflammatory cytokines and pain [6] and also that EPA may be beneficial in reducing the symptoms of DOMS and the level of inflammation associated with it.

In this potential therapeutic context, several studies have already queried whether omega-3/EPA doses between 300 mg/day to 2224 mg/day can affect the acute inflammation response and symptoms associated with DOMS after a single bout of exercise [3,20,21]. Lenn et al. [3], using 1800 mg/day of omega-3, reported that EPA had no effect on range of motion, pain, IL-6, TNF-α and creatine kinase levels. However, Phillips et al. [20] (using a daily cocktail of 300 mg of tocopherols plus 800 mg of docosahexaenoate plus 300 mg of flavonoids) and Bloomer et al. [21] (using 2224 mg/day of EPA) both reported a reduction in IL-6, CRP and TNF-α respectively, following a single bout of exercise. These studies in conjunction with the *in vivo *and *in vitro *work mentioned earlier [18,19] exemplify the confusion as to whether EPA may be beneficial in reducing pro-inflammatory cytokines linked with the inflammatory response and the symptoms associated with DOMS. To date the impact of fish oils on the acute and chronic response to a single bout of exercise remains unclear. Moreover, the conventional dose of 1000-2000 mg per day (of total fish oil or 180-360 of EPA) has mainly been far exceeded in the research to date.

### Aims and Objectives

The aims of the present study were therefore to investigate the effects of a dose of EPA supplementation just above standard recommendations, on basal inflammation, as well on both the acute and the chronic resistance exercise responses. It was hypothesised that not only could basal inflammation be decreased with EPA, but also that, despite a protocol designed to maximise potential DOMS, self-reported muscle soreness would decrease, circulating cytokine levels would be minimised, and muscle strength would be enhanced in the presence of EPA directly as a result of the creation of a more hospitable environment for muscle recovery.

## Materials and methods

### Pilot Study

A pilot study was conducted prior to testing to determine optimal joint angle and speed of contraction for maximal voluntary contractile efforts, whilst also testing for test-retest reliability both within and between sessions for quadriceps and hamstrings strength measurements. The pilot study revealed that the optimal angle and velocity for peak torque were 65° and 180°·s^-1 ^respectively for the selected population.

### Participants

A word-of-mouth advertising campaign was run within the local university campus. Forty convenience-sampled, non-smoking female university students responded to the call for participants. A further inclusion criterion was for participants to be currently taking progestin-only contraceptive pills and to be sedentary, in order to minimise the impact of intrinsic hormonal levels differences and/or variations in the habitual physical performance of the participants [22-24]. Other inclusion criteria were for participants to be naïve to resistance exercise, free from asthma, non-users of any vitamin/mineral supplementation (for at least two weeks prior to baseline). Participants also had to agree to maintain their habitual activity levels and to not commence a weight loss programme for the duration of the study (i.e. ~6 weeks). Exclusion criteria included drugs or alcohol abuse (two weeks prior to baseline), bacterial infection (two weeks prior to baseline), musculo-skeletal injury in the six months (preceding baseline) and use of anti-inflammatory and/or steroid medication (four weeks prior to baseline). Of the forty convenience sample twenty of the respondents (age 20.4 ± 2.1 years, body height 161.2 ± 8.3 cm and mass 61.48 ± 7.4 kg) fulfilled the inclusion criteria. All selected participants signed an informed consent form, approved by the local university ethics committee, prior to their inclusion in this study.

### Study Design

The study was a nine-week, double-blind placebo controlled design using the dietary supplement EPA versus lecithin as placebo. Participants were randomly allocated to receive either the EPA (N = 10) or the placebo (N = 10) supplementation for three weeks between baseline one (B1) and baseline two (B2). Participants were familiarised to all gymnasium and laboratory proceedings prior to B1. A week before B1 all participants were taken to the gym where one repetition maxima (1RM) were tested for the programmed exercises. Fasting venous blood samples, rating of perceived exertion (RPE), isometric and isokinetic strength assessments were then taken on four separate occasions including B1 (baseline 1), B2 (i.e. baseline 2 which occurred three weeks after baseline 1; B1 and B2 sessions were separated by three weeks of supplementation/placebo), S1 (i.e. resistance training session 1 which occurred post B2; here, one week after B2 participants performed a single bout of resistance training and were tested 48 hours after this bout of exercise), and finally S3 (i.e. resistance training session 3 which occurred after S1; here, upon completion of three weeks of weekly eccentric resistance training (including S1) participants were tested 48 hours after the final training session).

Three participants did not complete the entire experimental protocol resulting in data presented for EPA (N = 7) and placebo (N = 10). Participants were tested in the afternoon within the same two-hour window each day to minimise any impact of the circadian rhythm on the physical capacities of the participants [25].

### Supplementation

EPA supplementation was two 1000 mg softgel caps of omega-3, containing in total for the 2 gels 360 mg of EPA (18%) (MyProtein, Manchester, UK). This is twice the minimum dose as recommended by the American Heart Association. The placebo group received two 1000 mg softgel caps of lecithin (MyProtein, Manchester, UK). Participants were asked to take the capsules daily with a meal.

### Training Programme

**Figure 1:**
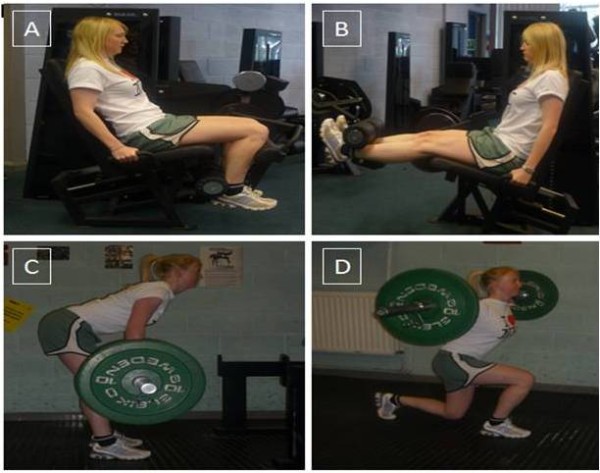
**Resistance exercise, A - leg flexion, B - leg extension, C - straight leg dead lifts, D - walking lunges (Authorised use of photos from a study participant, personal communication, April 26 2010)**.

Training intervention took place between 14:00 - 18:00 in an attempt to ensure optimal muscle performance [26,27] and thus potentially maximise DOMS. Upon completion of appropriate warm up, participants completed four exercises (See Figure 1) including walking lunges (with free weights), straight leg dead lifts (with free weights), leg extension (with a leg-extension machine; Pulse 562E class 's' 8/88. Pulse-fitness, Congleton, England), and leg flexion (with a leg flexion machine; Pulse 562E class 's' 8/88. Pulse-fitness, Congleton, England). Participants 1RM was pre-determined at the beginning of each training session, after which participants completed three sets of ten repetitions once a week working at 70% of their pre-determined 1RM over 45 minutes. Each repetition was completed within six seconds including concentric, isometric and eccentric phases. With regards to the progression of loading during training, for all three resistance training sessions (i.e. at S1, one week after S1 and at S3) participants' 1RM (for each of the four exercises) was determined at the beginning of the session. Participants then worked at 70% of the newly determined 1RM, thereby ensuring a load progression relative to the preceding training session. Thus, overall, each training session lasted 60 minutes including 1RM assessments and 3 sets of 10 repetitions of each of four exercises. This was similar to a protocol used elsewhere in previous research [28], designed to ensure muscle damage would occur.

### Strength Assessment

Maximal isometric and isokinetic (concentric and eccentric) knee extension were measured on the right leg for all participants. Measurements were assessed at 65°, and 180°·s^-1 ^as these were optimal knee angle and velocity for peak torque as demonstrated during the pilot study (full knee extension = 0°). Participants were seated on the isokinetic dynamometer (Cybex; Phoenix Healthcare Products, Nottingham, UK), which was calibrated prior to testing. The right knee was positioned so that the *epicondylus laterallis *was aligned to the centre of rotation of the motor arm. Straps were then positioned across the shoulder/chest, and over the right thigh to prevent any extraneous movement. Force application against the lever arm of the dynamometer was carried out with placement of the appropriate attachment set at a relative 80% of the lower leg length distally from the lateral condyle of the tibia. Participants were permitted a warm-up, which included five sub-maximal repetitions of knee flexions and extensions of the right limb at 100°·s. Testing included three trials, with 2 minutes rest between efforts, for both isometric and isokinetic conditions with peak knee extension torque used as the participant's strength score. Both visual and auditory feedback were used to encourage maximal efforts.

### Blood Collection and IL-6 detection

Participants fasted for eight hours prior to blood samples being taken from the anticubital vein of the forearm by a trained phlebotomist using a 21 ml gauge needle (S-Monovette, Sarstedt, Germany). Five millimetres of blood were taken and allowed to clot whilst standing for one hour on ice. The samples were then centrifuged (Hermle Z 380, Huddersfield) in 5°C at 4000 RPM for 10 minutes to separate the serum from the blood cells. Two aliquots (~900 μl each) of the resulting sera samples were taken and stored at -20°C for later analysis. IL-6 (R&D Systems inc. Minneapolis, USA. Sensitivity < 0.7 pg/ml; Intra-assay variability of 2.6%) concentrations were quantified using a standard ELISA (enzyme linked immuno sorbant assays) procedure.

### Statistical Analyses

Data were analysed using the Statistical Package for the Social Sciences (SPSS, Chicago, IL) version 18. The data on strength, IL-6 levels and changes in circulating IL-6 relative to baseline fulfilled the criteria for parametricity. IL-6 levels and relative changes (i.e. T1 = B2-B1/B1, T2 = S1-B1/B1 and T3 = S3-B1/B1) as well as strength data were analysed using a mixed design repeated measures two-way analysis of variance (ANOVA). The 'Within' factor was the protocol phase which had four levels (B1, B2, S1 and S3) and the 'between' factor was the treatment group with two levels (EPA treated vs. placebo). *Post hoc *tests were conducted with appropriate Bonferonni corrections. RPE data, as it was non parametric, was analysed within groups using a Friedman's test, followed by Wilcoxon signed-rank post-hoc tests. Between groups comparisons of RPE data were run using the Kruskal-Wallis test with Mann-Whitney post-hoc comparisons. An ANCOVA test (with Greenhouse-Geisser adjustments for non-equal variance) was used to evaluate whether the statistical significance of the changes in IL-6 since IL-6 levels were heterogeneous between groups at baseline (i.e. an unpaired student t-test showed that IL-6 in EPA and Placebo groups was significantly different at B1, P = 0.012). Evaluation of any association between IL-6, strength measurements (isometric and isokinetic) and RPE Borg pain scale were analysed using correlations and a multiple linear regression. Data are presented as mean ± standard error of the mean (SEM). Differences were considered significant at an alpha level of 0.05 (i.e. P ≤ 0.05).

## Results

Mean coefficient of variance (CV) for repeated measurements (intra-day variability) ranged between 1.0-2.0% and 0.8-2.7% on days one and two respectively for isometric measurements. The intra-day CV for the isokinetic measurements ranged from 1.3-1.9% and 1.4-2.7% on days one and two respectively. The inter-day CVs for repeated measurements ranged between 1.5-1.75% for isometric measurements, and 1.6-2.1% for isokinetic measurements.

### Isometric Strength

**Figure 2:**
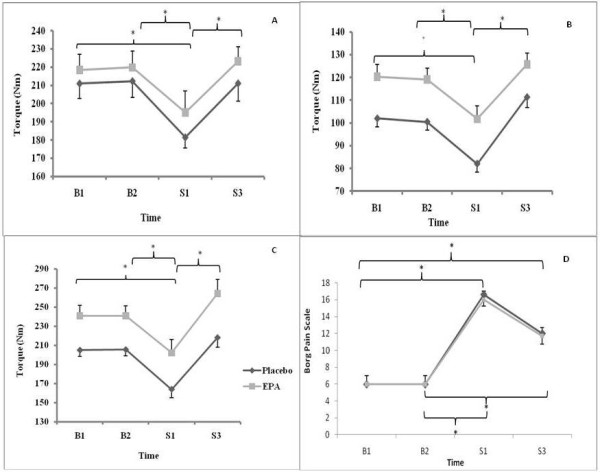
**EPA and placebo group changes in isometric (A) concentric (B) eccentric torque (C) and RPE pain scale (D) at B1 (1^st ^baseline), B2 (2^nd ^baseline i.e. after three weeks of supplementation), S1 (after one bout of eccentric exercises) and S3 (after three bouts of eccentric exercises)**. Data are mean ± SEM. * indicates significant difference (P ≤ 0.05).

There was a reduction in torque (see Figure 2A) of 13% (P = 0.007) between B1 (EPA 219 ± 34 Nm; placebo 211 ± 36 Nm) and S1 (EPA 195 ± 46 Nm; placebo 181 ± 23 Nm), and a 14% (P = 0.004) reduction in torque between B2 (EPA 219 ± 36 Nm; placebo 212 ± 35 Nm) and S1 (EPA 195 ± 46 Nm; placebo 181 ± 23 Nm). However, there was a 15% (P = 0.001) increase in the torque generated between S1 (EPA 195 ± 46 Nm; placebo 181 ± 23 Nm) and S3 (EPA 223 ± 32 Nm; placebo 211 ± 39 Nm) for grouped data. The main effect for groups shows that when all of the isometric strength for the EPA group was compared with the placebo group (EPA 214 ± 12 Nm vs. placebo 204 ± 15 Nm), they were not significantly different (P > 0.05). Thus, no interaction existed between treatment and time (P > 0.05).

### Concentric & Eccentric Torque

With concentric torque (see Figure 2B), there was a main effect of time for pooled data between B1 (100 ± 32 Nm) and S1 (94 ± 30 Nm) P = 0.008, B2 (101 ± 31 Nm) and S1 (94 ± 30 Nm) P = 0.018 and S1 (94 ± 30 Nm) and S3 (110 ± 34 Nm) P = 0.001. There was however no main effect of group (EPA 116 ± 7 Nm vs. placebo 91 ± 9 Nm, P > 0.05). There was no interaction between treatment and time in terms of concentric strength data (P > 0.05).

Similarly for eccentric torque (see Figure 2C), there was a main effect of time for pooled data between B1 (205 ± 65 Nm) and S1 (167 ± 63 Nm) P = 0.001, B2 (206 ± 64 Nm) and S1 (167 ± 63 Nm) P = 0.001 and S1 (94 ± 30 Nm) and S3 (222 ± 78 Nm) P = 0.001, but not for group (EPA 236 ± 22 Nm vs. placebo 174 ± 19 Nm, P > 0.05). There was thus no interaction between treatment and time in terms of eccentric strength (P > 0.05).

#### Muscle Soreness

There was no change in background pain scores (See Figure 2D) between the two baselines (B1 = 6.00 ± 0.00 and B2 = 6.00 ± 0.00, P > 0.05). Throughout the experimental phase, there was a non-significant trend for the placebo to demonstrate slightly larger ratings of perceived exertion (B1 = 6.00 ± 0.00, B2 = 6.00 ± 0.00, S1 = 16.62 ± 1.35 and S3 = 12.01 ± 1.25; P > 0.05) in comparison with the EPA group (B1 = 6.00 ± 0.00, B2 = 6.00 ± 0.00, S1 = 16.02 ± 0.82, S3 = 11.80 ± 1.11; P > 0.05).

### Cytokines

**Figure 3:**
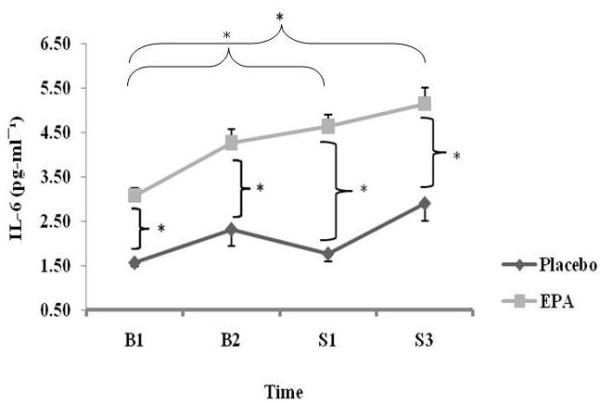
**Changes in IL-6 mediated inflammation for EPA and placebo groups for B1 (1^st ^baseline), B2 (2^nd ^baseline i.e. after three weeks of supplementation), S1 (after one bout of eccentric exercises) and S3 (after three bouts of weekly eccentric exercises)**. * indicates a significant difference (P ≤ 0.05). A repeated measures ANCOVA shows a significant (P = 0.002) main effect of time (differences between B1 to S1, and B1 to S3) as well as an interaction between time and group (P = 0.020). Data are mean ± SEM.

In the analysis of the IL-6 data (See Figure 3), since the study population was heterogeneous at baseline, this baseline difference therefore had to be partialled out. After accounting for the baseline differences in IL-6 levels, there was not only a main effect of time (i.e. experimental phase) on circulating IL-6 levels (P = 0.002), but there was also an interaction between time (B1, B2, S1, S3) and group (EPA vs. Placebo). In fact, the IL-6 levels in the EPA group, even after adjusting for baseline differences, were more augmented with exercise compared with levels in the absence of this treatment (relative to B1, the increments at S3 were 80 ± 26% in the placebo group, and 103 ± 60% in the EPA group; P = 0.020).

### Evaluation of bivariate associations

At day one (i.e. B1) and day twenty-one (i.e. B2), there were significant associations between isometric, eccentric and concentric strength only (r = 0.668 (isometric vs. concentric), r = 0.635 (isometric vs. eccentric), r = 0.802 (concentric vs. eccentric); p < 0.01 at B1 and (r = 0.688, r = 0.624, r = 0.790; p < 0.01) at B2). IL-6 level was not associated with any strength measure. RPE was constant across the population so no association could be computed. At days twenty-three (i.e. S1) and forty-four (i.e. S3), there was still a significant association between isometric, eccentric and concentric strength (r = 0.752, r = 0.819, r = 0.845; p < 0.001 at S1; r = 0.861, r = 0.797, r = 0.901; p < 0.001 at S3). IL-6 level was still not associated with any strength measure (P > 0.05). RPE, though now varying between participants, still showed no association either with strength measures or IL-6 levels (P > 0.05).

In addition, when a multiple linear regression was run on the pooled data with RPE scale as the dependent variable and IL-6, isometric/concentric/eccentric strength measures as independent variables, it was found that the stable model for predicting RPE scale (as the common indicator of DOMS) reads as, RPE scale = (-0.044 × isometric strength) + (0.137 × concentric strength) + (-0.049 × eccentric strength) + 4.074, r = 0.451, p = 0.002. Indeed IL-6 was not a good predictor of RPE scale.

## Discussion

Evidence from clinical and experimental studies suggests that omega-3 has a protective effect against cancer-induced cachexia, ageing-related chronic inflammation and other inflammatory diseases associated with excessive levels of cytokines [17]. This has led to further research to investigate whether EPA can have the same positive response on pro-inflammatory cytokines and symptoms associated with DOMS following exercise. Phillips et al. [20] and Bloomer et al. [21] both provided evidence to support the earlier *in vivo *and *in vitro *work [18,19], although both studies only observed the initial acute response after a single bout of exercise. These studies provided the basis for the current study in an attempt to observe if a dose of EPA which is twice the daily recommended level (i.e. ~2 × 180 mg per day) would inhibit acute and chronic IL-6 mediated inflammation, muscle soreness and RFGC following resistance exercise. The findings from the present study suggest that after three weeks of treatment, the standard dose of EPA may not be beneficial in ameliorating the symptoms associated with DOMS and IL-6 mediated inflammation response to exercise. In fact, the data would suggest that whereas strength and pain sensations related to resistance exercise are no different with/without EPA, exercise-induced IL-6 levels are in fact significantly elevated following three weeks of daily intake of EPA.

Babcock et al. [29] previously suggested two possible mechanisms that may be responsible for the anti-inflammatory ability of EPA. An initial response is for the EPA to be readily incorporated into the cellular membrane, where it alters linolenic and linoleic acids, which are essential for the production of arachidonic acid, the latter which is in fact involved in pain and inflammation. This was based on the earlier findings of Endres et al. [30], who looked at inflammation at a more cellular level in humans and rodents. They demonstrated that once within the cellular membrane, inflammation is affected by reducing prostaglandin E2 (PGE2) levels. Additionally a further mechanism was demonstrated by Lo et al. [31], who indicated that EPA modulates inflammation at a molecular level by down regulating the ubiquitin-proteasome proteolytic pathway, through decreasing translocation of nuclear factor-*κ*b (NF*κ*b). The authors indicated that EPA possesses the ability to reduce NF*κ*b, which is involved in protein degradation. A reduction in NF*κ*b would enable a positive environment for protein synthesis for repair of muscle following exercise, rather than a catabolic one. These are only two of the possible mechanisms by which EPA may alter levels of inflammation, however if effective then individuals in a clinical or sporting context may benefit. If either is effective at reducing inflammation associated with muscle damage, then it may be reasonable to assume that IL-6 mediated inflammation and DOMS would be reduced in the EPA group. However, the findings from the present study do not support this hypothesis.

DOMS post exercise is associated with RFGC, [7] muscle soreness [3] and elevated levels of cytokines [13]. The protocol used in the present study was designed to initiate an IL-6 mediated inflammatory response, muscle soreness and a RFGC, to demonstrate DOMS was achieved. Participants' pain was assessed 48 h post resistance exercise, and in accordance with previous research [3,20] muscle soreness did not alter between B1 and B2, however it did increase from B2 by 64% and 50% to S1 and S3 respectively (See Figure 2D). Participant's maximal isometric force ability decreased 48 h post resistance exercise by ~14% between B1 and S1, and B2 and S1. The reduction in participant's ability to generate force highlighted in the present study post resistance exercise is in accordance with previous research [2,16]. This reduction in participant's ability to generate force was matched by an increase in pain, which is in agreement with the work of Graven-Nielsen et al. [7]. The initial force reducing capacity of the muscles was evident in all three forms of contractions; however both forms of isokinetic contractions (concentric and eccentric) reported an increase between B1 and S3. A possible explanation for poor development in muscle force generating capacity for isometric contractions may have been due to the difference between the angles achieved when exercising compared to those used when strength assessments were carried out. When assessing muscle force generating capacity for isometric contractions the angle was set at 65°, however when performing resistance exercise this angle may have only been briefly achieved during the leg extension/flexion exercise (See Figure 1A and 1B). Morrissey et al. [32] reported an increase in motor unit activation at specified angles when working isometrically, therefore if the legs were not trained specifically at 65° degrees then there will be no increase in force generating capacity at that specific angle.

There was an increase in IL-6 48 h post resistance exercise of 26% and 43% between B1 and S1 and B1 and S3 respectively for grouped data. In addition there were also increases in IL-6 of 22% and 40% for grouped data between B2 and S1, and B2 and S3 respectively. These alterations in IL-6 are consistent with previous research demonstrating increases in IL-6 following an exercise protocol aimed at maximising DOMS (See Figure 3) [9,20]. The above support the assertion that the protocol used in the present study was effective at initiating DOMS.

In accordance with previous research eccentric contractions were included using a large muscle mass to maximise the IL-6 response [33]. Both EPA and placebo groups had an increase in IL-6, in agreement with previous research [2]; however, the increment in the EPA group was significantly greater than that in the placebo group.

Our findings of elevated IL-6 post-exercise contradict the previous research of Phillips et al. [20] and Bloomer et al. [21], who demonstrated a reduction in cytokines IL-6 and TNF-α 48 h post exercise. It should however be noted that Phillips et al. [20] used a combination of EPA, docasahexaenoate (DHA), tocopherols and flavonoids, and Bloomer et al. [21] used EPA and DHA in the supplement groups. This therefore raises the question of whether it was this combination of fish oils, or whether it was EPA, DHA, tocopherols or flavonoids, which were individually responsible for the reduction in IL-6, TNF-α and CRP. The variability of the fish oil used may be a possible explanation for the discrepancy between the findings of Phillips et al. [20] and Bloomer et al. [21] and the findings of the present study.

As mentioned above, the IL-6 response post exercise appears to be associated with greater generated torques [14] and muscle soreness post resistance exercise [3]. Notwithstanding the data from Lenn et al. [3] it is unclear whether there is a direct link between IL-6 and muscle soreness experienced post resistance exercise. The work of Graven-Nielsen et al. [7] demonstrated that muscle soreness significantly reduces MVC, possibly due to cytokines, such as IL-6 affecting nerve endings and activating nocieoceptors [6]. Therefore if IL-6 is associated with pain, then any reduction in IL-6 through EPA supplementation should be reflected in a reduction in pain. This, however, was not the case in the present study. In fact, our data show no association between IL-6 and any of the generally accepted markers of DOMS. The lack of any clear link between IL-6 and pain sensation is evidenced in data provided by Phillips et al. [20] which suggests that whilst a fish oil-treated group had a significantly reduced IL-6 level 72 h post exercise, this was not matched with a reduction in perceived pain. The data provided both here and in Phillips et al. [20] suggest that IL-6 may not be involved in the muscle soreness experienced post resistance exercise, and that other pro-inflammatory cytokines such as TNF-α or IL-1β may be responsible, however this was beyond the scope of the current study to determine and requires further research.

The data from the present study agrees with the findings from Lenn et al. [3], who suggested that EPA may not be beneficial at ameliorating the effects of DOMS and reducing levels of IL-6. However, this may have been due to any factor from insufficient work done to initiate a cytokine response (these authors trained a relatively small muscle mass (biceps brachii) and reported no increase in IL-6 post-exercise [14]), to the timing of blood sampling (24 h instead of 48 h post exercise [14]), or indeed EPA dosage (these authors did not specify the amount of EPA given to study participants), or a combination of both.

Although diet standardisation is notoriously difficult to monitor [34] this would allow researchers to truly assess the impact of EPA. Caughey et al. [35] ran a study involving four weeks of a diet high in cooking oils and spreads, followed by four weeks of fish oil capsules (a daily intake of 1620 mg of EPA (i.e. 78% more than the dose used in the present study) and 1080 mg of DHA). The authors reported significantly inhibited basal TNF-α and IL-1β synthesis.

In the current study blood samples were taken 48 h post resistance exercise however, both conflicting and supporting evidence exists for peak release of IL-6 during this time period. Hellsten et al. [36] used a protocol similar to that of the present study with blood samples ranging from one to 96 h post exercise. The authors suggested that the prolonged release of IL-6 may be due to the increase in cellular xanthine oxidase activity. Furthermore, Pedersen et al. [14] indicated that IL-6 acts as an intracellular signaller for leucocytes, such as neutrophils, which migrate towards chemoattractants, such as IL-6. These neutrophils then accumulate at the site of muscle damage, where the lifespan is between 24-48 h, suggesting a possible explanation for peak IL-6 48 h post exercise. Yet evidence to the contrary of the two aforementioned authors was provided by Croisier et al. [8] and Steensberg et al. [37]. Both studies indicated that IL-6 peaks within the first 30 minutes to six hours post exercise, prior to returning to baseline values. Peak IL-6 levels were reported by Croisier et al. [8] and Steensberg et al. [37] as 10 pg/ml and 8 ng/l, respectively. Both studies used protocols similar to that of the present study, although the peak levels of IL-6 were not consistent with the present study of 4.6 pg/ml. It should be pointed out here that Steensberg et al. [37] took muscle biopsies, therefore a direct comparison with the present study cannot be made. Steensberg et al. [37] indicated that the main function of the early release of IL-6 is to operate in a 'hormone-like manner' and play a role in carbohydrate metabolism, through activating extramuscular substrates and supplementing substrate delivery during and post resistance exercise. Furthermore, this hormone-like behaviour of IL-6 stimulates the hypothalamic pituitary axis (HPA) axis, and in doing so contributes to the inflammatory response post exercise. Moreover Al-Shanti et al. [17] demonstrated that early release of IL-6 has beneficial effects on skeletal muscle cells since adding IL-6 to myoblasts enhanced cell proliferation in a linear fashion, with peak cell count occurring within the first 24 h. Supporting the work of Steensberg et al. [37], Febbario et al. [16] suggested that the early release of IL-6 is involved in hepatic glucose metabolism, as IL-6 has been shown to inhibit glycogen synthase activity and accelerate glycogen phosphorylase activity. These findings suggest that IL-6 is involved in mediating blood glucose homeostasis, when skeletal muscle increases its uptake of blood glucose.

In the present study, despite being non-significant, the EPA group had a greater increase in isometric and isokinetic eccentric torque generation between B2 and S3 compared to the placebo group (2.23 and 10%, 0 and 6%, respectively), and these were associated with greater IL-6 levels increases compared with the placebo group. These findings could provide some indirect support to the in-vitro work of Al-Shanti et al. [16] and the in-vivo research of Xing et al. [12], who reported that IL-6 is beneficial in promoting muscle growth and repair, and is essential for controlling local and systemic inflammatory response. Therefore it is possible that the elevated levels of IL-6 in the EPA group may have been linked to a relatively enhanced muscle contractile capacity (as shown through higher strength increments), resulting in greater glycogen depletion, which would then cause an increase in glucose metabolism as well as an increase in circulating IL-6 levels. Whatever the case, the underlying mechanism of how EPA impacts on the production of IL-6 is unclear and requires further research.

## Conclusion

Based on the protocol used in the present study the data suggests that a 360 mg daily intake of EPA over three weeks may not be beneficial in reducing DOMS or IL-6 mediated inflammation, at least not in the way we would have expected it to. In fact it would appear that this dose enhances the exercise-induced cytokines surge by a factor of ~20%.

Further research may include varying levels of EPA supplementation, as Babcock et al. [29] suggests there may be a dose-response relationship of EPA on the inhibiting effect on IL-6 production. In addition it may be interesting to observe other pro-inflammatory cytokines such as IL-1, IL-8 and TNF-α as indicators of inflammation caused by muscle damage, and the interactions if any, that EPA may have with them. Furthermore the present findings suggest that the temporal expression of IL-6 requires further investigation.

## Competing interests

The authors declare that they have no competing interests.

## Authors' contributions

DH, as post-graduate student, was responsible for recruiting the study participants, applying the study intervention, recording the data and writing the first draft of the manuscript. GLO, as his director of study developed the idea, trained DH in the laboratory skills, helped with the statistical analyses and refined the final version of the manuscript. Both authors read and approved the final manuscript.
